# Implementing evidence-based recommended practices for the management of patients with mild traumatic brain injuries in Australian emergency care departments: study protocol for a cluster randomised controlled trial

**DOI:** 10.1186/1745-6215-15-281

**Published:** 2014-07-11

**Authors:** Marije Bosch, Joanne E McKenzie, Duncan Mortimer, Emma J Tavender, Jill J Francis, Sue E Brennan, Jonathan C Knott, Jennie L Ponsford, Andrew Pearce, Denise A O’Connor, Jeremy M Grimshaw, Jeffrey V Rosenfeld, Russell L Gruen, Sally E Green

**Affiliations:** 1 Department of Surgery, Central Clinical School, Monash University, Melbourne, Australia; 2 National Trauma Research Institute, The Alfred & Monash University, Melbourne, Australia; 3 School of Public Health and Preventive Medicine, Monash University, Melbourne, Australia; 4 Centre for Health Economics, Monash University, Melbourne, Australia; 5 School of Health Sciences, City University London, London, UK; 6 Melbourne Medical School, The University of Melbourne, Melbourne, Australia; 7 Department of Emergency Medicine, Royal Melbourne Hospital, Melbourne, Australia; 8 Monash-Epworth Rehabilitation Research Centre, Epworth Hospital, Melbourne, Australia; 9 School of Psychology and Psychiatry, Monash University, Melbourne, Australia; 10 MedSTAR Emergency Medical Retrieval Service, Adelaide, South Australia; 11 Royal Adelaide Hospital Emergency Department, Adelaide, Australia; 12 Clinical Epidemiology Program, Ottawa Hospital Research Institute, Ottawa, ON, Canada; 13 Department of Medicine, University of Ottawa, Ottawa, ON, Canada; 14 Department of Neurosurgery, The Alfred Hospital, Melbourne, Australia; 15 Department of Trauma, The Alfred Hospital, Melbourne, Australia

**Keywords:** Mild traumatic brain injury, Cluster trial, Emergency department

## Abstract

**Background:**

Mild head injuries commonly present to emergency departments. The challenges facing clinicians in emergency departments include identifying which patients have traumatic brain injury, and which patients can safely be sent home. Traumatic brain injuries may exist with subtle symptoms or signs, but can still lead to adverse outcomes. Despite the existence of several high quality clinical practice guidelines, internationally and in Australia, research shows inconsistent implementation of these recommendations. The aim of this trial is to test the effectiveness of a targeted, theory- and evidence-informed implementation intervention to increase the uptake of three key clinical recommendations regarding the emergency department management of adult patients (18 years of age or older) who present following mild head injuries (concussion), compared with passive dissemination of these recommendations. The primary objective is to establish whether the intervention is effective in increasing the percentage of patients for which appropriate post-traumatic amnesia screening is performed.

**Methods/design:**

The design of this study is a cluster randomised trial. We aim to include 34 Australian 24-hour emergency departments, which will be randomised to an intervention or control group. Control group departments will receive a copy of the most recent Australian evidence-based clinical practice guideline on the acute management of patients with mild head injuries. The intervention group will receive an implementation intervention based on an analysis of influencing factors, which include local stakeholder meetings, identification of nursing and medical opinion leaders in each site, a train-the-trainer day and standardised education and interactive workshops delivered by the opinion leaders during a 3 month period of time. Clinical practice outcomes will be collected retrospectively from medical records by independent chart auditors over the 2 month period following intervention delivery (patient level outcomes). In consenting hospitals, eligible patients will be recruited for a follow-up telephone interview conducted by trained researchers. A cost-effectiveness analysis and process evaluation using mixed-methods will be conducted. Sample size calculations are based on including 30 patients on average per department. Outcome assessors will be blinded to group allocation.

**Trial registration:**

Australian New Zealand Clinical Trials Registry ACTRN12612001286831 (date registered 12 December 2012).

## Background

Head injuries are a frequent presentation to emergency departments (EDs) worldwide. They are caused by external forces to the head (such as sport, falls, motor vehicle accidents, assaults or blast injuries) [1]. Country-based incident estimates range from 108 to 332 hospitalised new cases per 100,000 population per year [2], and the incidence is rising as a consequence of increased transport-related injuries in low- and middle-income countries [1]. Traditionally, head injuries are classified as mild, moderate or severe head injury, depending on the patient’s presenting level of consciousness as expressed by the Glasgow Coma Scale (GCS) score [3]. The scale ranges from a low of 3 (comatose) to 15 (awake and following commands). The vast majority of patients (80 to 90% depending on definitions) present with normal or near normal GCS scores (14-15/15) and are therefore classified as “mild”. In non-paediatric patients, the highest incidence of mild traumatic brain injury (mTBI) is seen in males between the ages of 15 and 24 years and in men and women 65 years of age and older [4].

In most cases, patients who experience mTBI will recover fully, typically within days to months. Up to 15% of patients with a normal GCS score of 15 have an acute lesion on head computed tomography (CT), but less than 1% of these patients have a lesion that requires neurosurgical intervention [4-6]. However, up to 15% of patients diagnosed with mTBI experience persistent disabling problems [7,8] including reduced functional ability, heightened emotional distress, and delayed return to work or school [9,10] 3 months or more after injury. As the ED is the main, and often only, point of medical contact for these patients, ED care may provide opportunities to impact on the outcomes of these patients.

The challenge for the emergency physician is to identify which patients with a head injury have an actual traumatic brain injury (TBI) requiring further management, and which patients can safely be sent home [4]. Patients with subtle symptoms or signs can still progress to suboptimal outcomes [4]. Despite the existence of several high quality evidence-based clinical practice guidelines (EBCPGs) internationally and in Australia [11], research shows inconsistent implementation of recommended practices. In earlier phases of our study, relevant EBCPGs were identified and assessed for their quality [11]. Subsequently, key clinical recommendations and the evidence underpinning these recommendations were studied, and two consensus meetings were organised to develop locally relevant recommendations to serve as the basis for developing our management outcome measures [12]. Table 1 outlines the evidence-based recommendations, their relevance to the management of this patient group, and evidence regarding gaps in practice.

**Table 1:** Key clinical recommendations in the management of mild traumatic brain injury in emergency departments

**Key recommendations and relevance to management**	**Research highlighting the ‘evidence-practice’ gap**
**Post-traumatic amnesia should be prospectively assessed in the emergency department using a validated tool**	
Post-traumatic amnesia (PTA) is defined as "an interval during which the patient is confused, amnestic for ongoing events and likely to evidence behavioural disturbance" [13]. It may manifest as repetitive questioning or short-term memory deficits [14] and has been shown to have better predictive ability with clinical outcomes compared with Glasgow Coma Scale (GCS) [15-18] (GCS assesses consciousness but not whether the patient is able to lay down new memories). Various validated tools to asses PTA are available such as the Revised Westmead PTA tool, or the Abbreviated Westmead PTA scale (A-WPTAS), the latter being an extended version of the GCS and specifically developed for use in the emergency department (ED). The A-WPTAS standardises some of the questions of the GCS and adds a memory test. Screening for PTA using such a validated tool may reduce the risk of failing to classify mild traumatic brain injury (mTBI) patients and prevents patients from being discharged from hospital while they are suffering from acute cognitive impairment [19,20].	A retrospective audit in two Australian EDs showed rates of assessment of PTA in adults (for those with an initial GCS of 14 or 15) as 0% (95% CI 0% to 14%; n = 24) in one hospital, and 31% (95% CI 24% to 39%; n = 164) for a second (which had a protocol in place) (Bosch M, McKenzie J, unpublished observations). We are not aware of any published studies reporting rates in adults.
**Guideline-developed criteria or clinical decision rules should be used to determine the appropriate use and timing of computed tomography imaging**	
The aims of using clinical decision rules to determine the need for a computed tomography (CT) scan are to ensure patients at risk of developing intracranial injuries receive a scan, and to decrease unnecessary scanning. Several clinical decision rules have been developed worldwide, some of which have been externally validated, most notably the Canadian Computed Tomography Head Rule (CCTHR) [21] and the New Orleans Criteria [22]. However, both these rules used loss of consciousness or amnesia as entry criteria, which means they could not be reliably used for all patients presenting to the ED with a head injury. Studies show that intracranial complications can also occur without loss of consciousness/PTA, particularly in the presence of other risk factors [23-25]. Therefore, more recent guidance has been developed that is applicable to all patients irrespective of presence or absence of loss of consciousness/PTA [4,14,26]. Most evidence-based clinical practice guidelines for the management of patients with mTBI have adapted one or more of these rules. They show considerable concordance [27]. The Institute for Trauma and Injury Management, New South Wales, developed simple recommendations for the identification of high risk patients based on the presence of single criteria that are applicable in the Australian setting [14].	It is difficult to establish target rates for appropriate CT scanning in patients with mTBI (that is, the percentage of mTBI patients who should receive a scan) because this is dependent on the case mix of patients (for example, hospitals that service an older demographic may (appropriately) have higher CT scanning rates for mTBI patients) and the leniency of the rules or guidelines. A study comparing percentages of scans that would be required by applying six different rules found rates between 50% and 71% [28]. A Canadian study using the CCTHR estimated a rate as low as 62.4% was possible and safe [29]. Estimates of how frequently appropriate decisions are made about the need for CT scanning (in populations with slightly variable definitions and using several different rules) range from 66% in the UK [30], 73% in Scandinavia [31], 82.5% in Canada [29], 80% and 92% in indigenous and non-indigenous Australians [32], and 65% to 91% in the US [33]. A study [34] surveying Australian ED physicians (response rate 54.2%, n = 417) showed that 82% had awareness of CCTHR but only 32% used it.
**Verbal and written patient information consisting of advice, education and reassurance should be provided upon discharge from the emergency department**	
Providing patients with information upon discharge serves two purposes: 1) to inform the family/carer about what to observe and what actions to take if the patient’s neurologic condition deteriorates significantly after discharge from the ED [35]; and 2) to provide information regarding post-concussive symptoms, symptom management, and prevention of future head injuries [36-39]. A randomised controlled trial [40] of 202 adults with mTBI in Australia evaluated the impact of patient information (booklet) on outcomes. The booklet outlined common symptoms associated with mTBI, their likely time course and suggested coping strategies. By 3 months, the intervention group had lower scores on most items on a post-concussion checklist, significantly so for anxiety (*P* < 0.04) and sleeping difficulty (*P* < 0.01). They also had lower scores on a ‘global severity’ score. There was no statistical difference between the groups on formal neuropsychological assessment.	Studies show that a large proportion of mTBI patients do not receive written information upon discharge from the ED, ranging from 36% [41] and 51% [30] in the UK, to 63% in the US [42]. Studies looking at the quality and content of discharge pamphlets [35,37,38,43] found that the information regarding post-concussive symptoms and reassurance is missing in 41% [37] to 60% [38] of pamphlets reviewed. One study surveyed nurses (25% response rate) regarding their teaching habits [36], and concluded that in general they were more focused on providing injury-specific information and less on mTBI, symptom management or strategies to prevent future brain damage.

We are not aware of trials which have evaluated implementation of this suite of recommendations in ED practice internationally or in Australia. A cluster randomised trial (CRT) performed by Stiell and colleagues [29] in Canada implemented the Canadian CT Head Rule (CCTHR) to reduce unnecessary CT scans. Their implementation intervention package included education (distribution of articles describing the development and validation of the rule, pocket cards and posters, and a 1-hour teaching session to review the evidence and clinical application of the rule), and a mandatory real-time reminder of the rule at the point of CT scan request that required checking off the rule before the scan would be performed by the imaging department [29]. The intervention was designed to be low-cost and simple and had been shown to be effective in reducing cervical spine imaging rates for neck trauma in a simultaneous study [44]. In the implementation of the CCTHR, the intervention failed to reduce the number of CT scans requested; in fact, CT imaging rates increased in both intervention and control group (62.8% before compared with 76.2% after, absolute difference +13.3%, and 67.5% to 74.1%, absolute difference +6.7%, respectively; a difference in change between groups of 6.6%). However, the baseline rates were much lower than expected and approaching the estimated ‘safe’ rate (62.4%), which may have reduced the likelihood of further reductions (that is, indication of a ceiling effect) [29].

Increasingly it is considered best practice to base interventions aiming to change clinical practice on a theoretical approach to identifying factors potentially influencing practice change [45]. Theory can offer a generalisable framework for considering effectiveness across different clinical conditions and settings [46], and, given the wide variety of factors that may influence practice change, basing interventions on different theoretical assumptions may prevent overlooking important factors [47] and will ultimately help to understand how and why change is achieved. Additionally, this approach offers the possibility of optimising interventions to increase effect sizes in subsequent studies.

### Aims and objectives

The aim of this trial, which is part of the Neurotrauma Evidence Translation (NET) programme [48], is to test the effectiveness of a targeted, theory- and evidence-informed implementation intervention to increase the uptake of three key clinical EBCPG recommendations regarding the management of adult patients (18 years of age or older) who present to Australian EDs with mild head injuries, compared with passive dissemination of the EBCPG.

More specifically, the primary objective is to establish whether the intervention is effective in increasing the percentage of patients for which a prospective measure of post-traumatic amnesia (PTA) using a validated tool is performed in the ED until a perfect score is achieved or the patient is transferred or admitted.

Secondary objectives include establishing whether the intervention is effective in: increasing the percentage of patients for which two other assessment methods of PTA were performed (assessment without using tool, and whether the administration of the validated tool was completed at least once); increasing the percentage of patients for which CT scanning is appropriately performed; increasing the percentage of patients who receive patient information upon discharge home from the ED; changing factors that are thought to mediate the effect of the intervention (such as increasing the staff members’ knowledge, or decreasing negative beliefs about consequences); and improving selected patient clinical outcomes and quality of life.

In addition, our study aims to: quantify the trade-off between the hypothesised improvement in patient management and patient health outcomes, and the additional costs (savings) arising from delivery of the implementation intervention and from any subsequent changes in clinical practice and healthcare utilisation by conducting a cost-effectiveness analysis; and evaluate intervention fidelity, assess acceptability of the intervention, and assess perceptions around success of the implementation processes and influencing factors, by conducting a process evaluation.

## Methods/design

**Figure 1:**
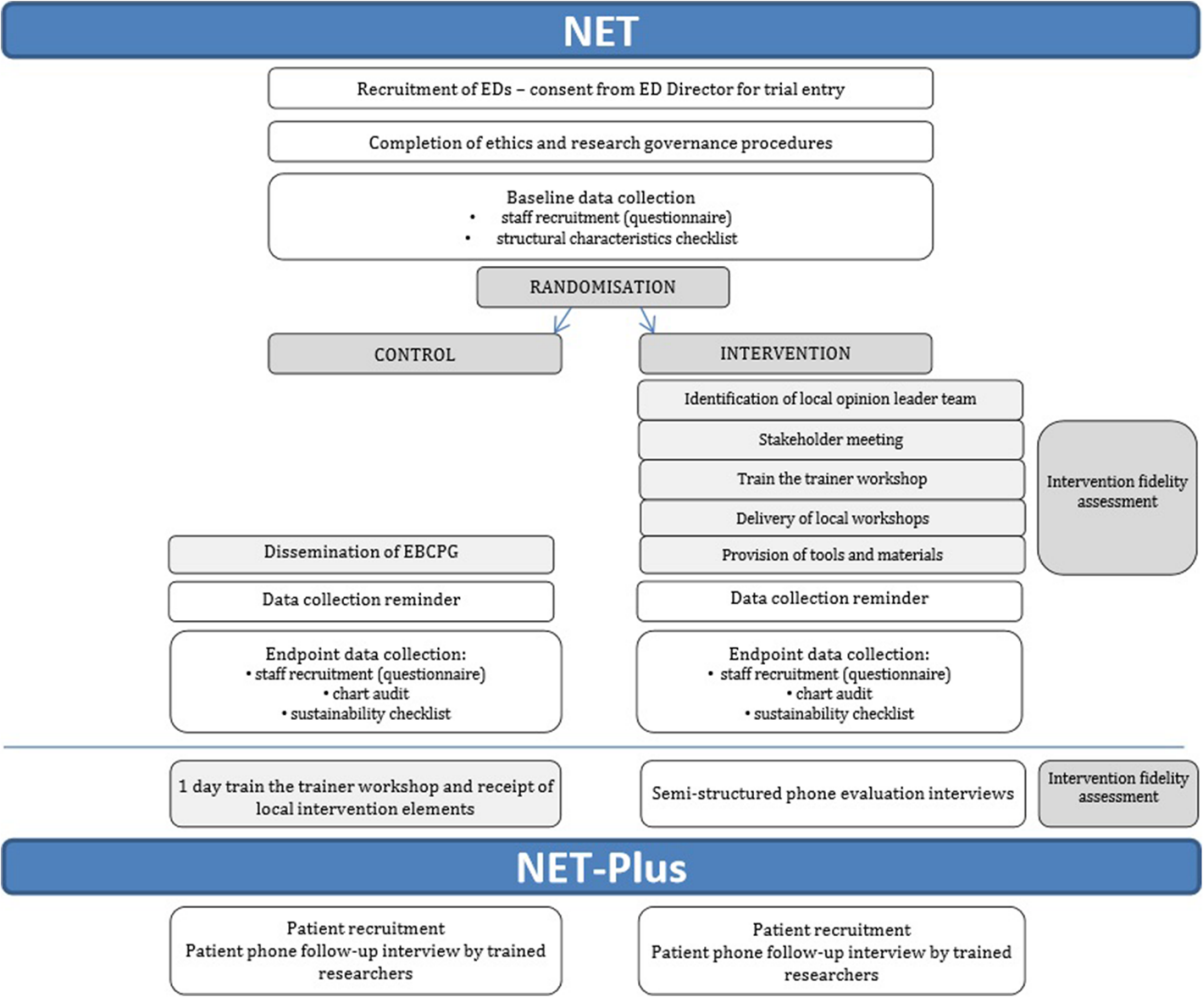
**Trial design.** EBCPG, evidence-based clinical practice guideline; ED, emergency department; NET, Neurotrauma Evidence Translation.

The design of this study is a CRT, with the EDs being the clusters including the ED staff members involved in the treatment of patients with mTBI and the patients treated. A randomised design is the preferred one to evaluate the effectiveness of an intervention since it minimises bias in estimating intervention effects compared with other study designs [49,50]. In this study, clusters (hospital EDs) have been chosen for two reasons: the intervention is targeted at the team of ED staff, and EDs represent patient populations in geographical areas, precluding the use of an individually randomised design [51,52].The study has two levels of participation (Figure 1): NET and NET-Plus. NET will measure clinical practice outcomes, but not patient outcomes, whereas NET-Plus will include both. These two options will be offered to hospitals because we anticipate that some may prefer not to recruit patients.

### Recruitment and eligibility

#### Recruitment of emergency departments and inclusion/exclusion criteria

The sampling frame for this study will be derived from the Australasian Society for Emergency Medicine ED Directory list of 24-hour Australian EDs (regional and metropolitan) [53]. EDs involved in the pilot and/or development of the intervention will not be invited to participate in the trial.

Hospitals will be included if we receive written informed consent from the ED Director to randomise the ED to control or intervention group. Hospitals will be excluded if they are either non-24-hour EDs or specialised hospitals (for example, women’s or children) and do not therefore routinely treat adult patients with TBI. In addition, EDs will be excluded if they do not have a CT scanner on site, or if there is significant risk of bias (for example, risk of contamination due to two EDs having the same ED Director, or senior influential clinicians working across sites).

We intend to recruit 34 EDs (see sample size section). Based on a previous Australian study (Knott J, Bosch M, personal communications) which observed a 50% ED participation rate, we plan to initially invite 68 randomly selected hospitals to participate in the trial. Our recruitment strategy will follow recommendations regarding the recruitment for CRTs in secondary care settings [52]. ED directors will be considered ‘gatekeepers’ (people who can grant formal permission for the organisation to be involved) of the clusters. As a first step they will be contacted by telephone to inform them about the study, and let them know that they will receive a recruitment pack (including an invitation letter from the research team, explanatory statement, and an expression of interest form). The ED Director will be encouraged to discuss the trial with the relevant stakeholders within their ED (in particular Directors of Trauma or staff members responsible for trauma and/or research, and senior clinicians). Upon receipt of the expression of interest, a recruitment meeting will be organised (via telephone, skype, video-link or face-to-face) with the study investigators and the relevant stakeholders in the ED. During this recruitment meeting, the details and logistics of the study will be discussed. Following this meeting, the ED Director will be asked to provide written informed consent to trial entry (either NET or NET-Plus), using a consent form sent prior to the meeting [51,52,54]. Non-responders will be followed up by email and telephone [55] at least once, before inviting the next random selection of hospitals.

The content of the recruitment information will be designed to target factors influencing the decision to participate in this trial [56] that were identified in semi-structured interviews in an earlier phase of this study (for example, by providing information around resources and including information around the benefits for the patients to create ‘tension for change’). In addition, professional bodies will be engaged to endorse the trial [57].

#### Recruitment of emergency department staff members for data collection purposes and inclusion/exclusion criteria

ED medical and nursing staff will be invited to complete two questionnaires (at baseline and endpoint). The sampling frame for staff participants will be based on the active list of ED medical and nursing personnel in each ED. Inclusion criteria for nursing staff are: 1) current ED appointment; 2) on active practice roster; 3) enrolled or registered nurses. Inclusion criteria for medical staff are: 1) current ED appointment; 2) on active practice roster; 3) registrars, Hospital Medical Officers or consultants. For both groups, exclusion criteria are: 1) students/interns; 2) clinicians not currently engaged in clinical practice (for example, academic staff); and 3) for nurses, bank or agency nurses.

We plan to randomly select 50 eligible ED clinicians per clinical group per hospital (that is, 100 in total) when available at baseline and endpoint. For hospitals with fewer than 50 clinicians per clinical group, we plan to invite all clinicians satisfying the inclusion criteria to participate for data collection. Staff members who were contacted at baseline will be asked to participate at endpoint, and additional staff will be randomly selected in instances where staff have been lost to follow-up. Staff members will be included if they fit our inclusion criteria and consent to participate.

In addition, some key staff members in eight intervention sites will be recruited to participate in a brief evaluation interview over the telephone. To identify staff, we will use a key-informant method [58].

Finally, all ED Directors or their delegate will be invited to complete two brief checklists over the telephone regarding ED characteristics at baseline and endpoint.

#### Identification of study patients and inclusion/exclusion criteria

In order to determine the effect of the intervention on clinical practice outcomes, a chart audit will be conducted by independent chart auditors. Patients are eligible if they are aged 18 years or older, present to the ED within 24 hours following acute blunt head trauma, and have a GCS score of 14 or 15 at presentation [14]. Exclusion criteria include penetrating injuries, and non-traumatic TBI such as caused by stroke.

A patient identification protocol will be developed and chart auditors will be trained with the aim of maximising consistency in identification of patients, and minimising selective recruitment. We anticipate that patients will be identified retrospectively from clinical records using medical codes (for example, relevant International Statistical Classification of Diseases and Related Health Problems Tenth Revision, Australian Modification (ICD-10AM) discharge codes). Instructions will be piloted and tailored to the hospital medical record system. As there may be some between-hospital variation in the approach taken, the exact process to identify patients will be carefully recorded in each hospital.

#### Recruitment of patients for follow-up – NET-Plus only

In hospitals which choose to participate in NET-Plus, patients included in the chart audit will be contacted by telephone by an ED staff member to invite them to participate in a follow-up telephone interview questionnaire by trained researchers. Informed consent to pass on their contact details to the NET research team will be sought (see ethical review and informed consent).

For this component, additional exclusion criteria are: 1) not consenting to participate in the follow-up study; and 2) not being able to participate in a telephone interview, because of an inability to speak English, being hearing-impaired, or having cognitive or intellectual impairment (history of dementia, other neurological disorder, intellectual disability drug or alcohol abuse or other major psychiatric disorder such as psychosis for which hospitalisation has been required).

These criteria will be confirmed by the ED staff asking for consent and again by the trained researcher at the start of the interview.

### Randomisation and allocation concealment

EDs will be allocated to the intervention and control groups using the method of minimisation proposed by Pocock and Simon [59]. Minimisation has been shown through statistical simulation studies to outperform stratified allocation methods for maintaining baseline balance in prognostic factors [60]. In addition, the method allows for balancing across a greater number of prognostic factors. The minimisation factors will include: the existence of a protocol for mTBI (consistent with our recommendation for appropriate PTA assessment); size (annual presentation rate 2012), included as a proxy for other variables that are harder to measure such as staffing; rurality; and type of participation (NET or NET-Plus). Minimisation is essentially a deterministic method [60], and therefore use of this method can increase the risk of selection bias [61]. We plan to reduce the risk of selection bias through inclusion of a random component and allocation of batches of EDs by a statistician independent of the trial. The statistician will be provided with a file containing only ED codes and the minimisation factors (thus no identifying information). For each batch of EDs, the statistician will randomise the order of EDs before using the minimisation algorithm to allocate the EDs to intervention and control groups.

### Blinding

Due to the nature of the intervention, it will not be possible to blind ED staff members to group allocation. To limit the possibility of selection and detection bias, chart auditors will be independent, where possible, of the hospital. In instances where the hospital does not approve an independent chart auditor, a solution will be discussed which aims to reduce the risk of selectively identifying patients (for example, using a staff member from another unit who will receive training from the research team). External chart auditors and the trial statistician will be blinded to group allocation.

### Interventions

#### Intervention group

Intervention group EDs will receive a targeted, theory- and evidence-informed implementation intervention. The intervention builds upon the previous phases of this study. In phase one, relevant EBCPGs were identified, assessed for their quality, and the focus of the study was determined [11,12]. In phase two, semi-structured interviews underpinned by theoretical perspectives on individual and organisational change were conducted with 42 ED staff members in 13 Victorian EDs. Thematic analysis was used to identify the factors perceived to be influencing current practice as well as change in the ED setting. In phase three, our intervention was developed to address the modifiable influencing factors to maximise the effectiveness of the intervention. Intervention components were piloted for feasibility and acceptability. Table 2 summarises the content of the intervention. Only broad components are described in this protocol to prevent contamination of the control group. The process of development of the intervention and a description of its content will be published in detail post-data collection.

**Table 2:** Planned delivery of the intervention

*Intervention and control group*	
1.	An electronic/printed copy of *Initial Management of Closed Head Injury in Adults* guideline [14].
2.	Data collection reminder sticker/flag in system and education around the importance of documenting information for mild traumatic brain injury patients to optimise data collection.
*Intervention group only*	
3.	One hour face-to-face multidisciplinary stakeholder meeting in each participating hospital with key stakeholders (both clinical and change management) and senior Neurotrauma Evidence Translation (NET) clinicians and researcher to create buy-in at ‘organisational’ level for the changes by discussing the key recommendations and underlying evidence; discussing intervention components and how to overcome anticipated barriers in their implementation etc.
4.	Identification of multidisciplinary local opinion leader team (medical and nursing) via key-informant method [58] (emergency department Directors will be provided with a description of the types and characteristics of people suited to the role).
5.	One day train-the-trainer interactive workshop, led by content experts and senior NET clinicians, attended by the nursing and medical opinion leaders, consisting of information provision and skills training - both in relation to the key-recommendations as well as in relation to their role in the study.
6.	Delivery of materials for local workshops (brief 20 minute sessions) in relation to the key recommendations presented by the clinical opinion leaders to staff in their emergency department over a 3 month period of time.
7.	Provision of relevant tools and materials (for example, screening tools [14] and information booklets [40] translated into five languages that are commonly spoken in Australia).

#### Control group

Control EDs will receive a copy of the EBCPG [14] and all the materials/components needed for the outcome assessment. Post-data collection, control EDs will be invited to a repeat train-the-trainer 1-day interactive workshop where they will also receive the components for the local education and all other intervention materials.

#### Timing of recruitment, intervention delivery, and follow-up

Figure 1 shows the flow of an ED through the trial. We started recruitment and completion of ethics and research governance procedures in February 2013. Baseline data collection has commenced (October 2013). Intervention delivery and endpoint data collection will take place in 2014.

### Study outcomes and outcome measurement

Tables 3 shows our clinical practice outcomes and data collection methods.

**Table 3:** Clinical practice and proxy clinical practice outcomes and data collection methods

	**Method**	**Outcome assessment period/timing**	**Data source**	**Level data collected**	**Analysis level**
**Primary outcome**					
Appropriate post-traumatic amnesia screening (PTA)	Chart audit (retrospective)	2 month period post-intervention	Hospital record	Patient	Patient
**Secondary outcomes**					
***Clinical practice outcomes***					
PTA screening tool	Chart audit (retrospective)	2 month period post-intervention	Hospital record	Patient	Patient
Memory - clinical assessment	Chart audit (retrospective)	2 month period post-intervention	Hospital record	Patient	Patient
Computed tomography scan - clinical criteria (CT)^1^	Chart audit (retrospective)	2 month period post-intervention	Hospital record	Patient	Patient
Provision of patient information (INFO)	Chart audit (retrospective)	2 month period post-intervention	Hospital record	Patient	Patient
Safe discharge based on PTA and INFO	Chart audit (retrospective)	2 month period post-intervention	Hospital record	Patient	Patient
Safe discharge based on PTA, CT, and INFO	Chart audit (retrospective)	2 month period post-intervention	Hospital record	Patient	Patient
***Proxy measures of clinical practice***					
Self-report of adherence to recommended practice					
• PTA	Staff questionnaire (1-item)	Baseline/Endpoint	Doctors	Staff member	Staff member
			Nurses		
• CT	Staff questionnaire (1-item)	Baseline/Endpoint	Doctors	Staff member	Staff member
• INFO	Staff questionnaire (1-item)	Baseline/Endpoint	Doctors	Staff member	Staff member
			Nurses		
Behavioural simulation^2^ to adhere to recommended practice					
• PTA	Staff questionnaire (clinical vignettes)	Endpoint	Doctors	Staff member	Staff member
			Nurses		
• CT	Staff questionnaire (clinical vignettes)	Endpoint	Doctors	Staff member	Staff member
			Nurses		
• INFO	Staff questionnaire (clinical vignettes)	Endpoint	Doctors	Staff member	Staff member
			Nurses		

^1^Criteria that justify a scan are: age 65 years or older; GCS <15; amnesia; suspected skull fracture; vomiting and coagulopathy (see Additional file 1). ^2^Clinical decision in response to individual simulated patients, for example in patient scenarios.

#### Primary outcome

Our primary outcome is ‘appropriate PTA screening’ (PTA; Table 3). This outcome measures whether a prospective assessment of PTA was appropriately undertaken, where appropriately undertaken is defined as using a validated tool, until a perfect score was achieved (indicating absence of acute cognitive impairment) before the patient was discharged home (or the patient was admitted or transferred). This measure has been chosen as the primary outcome for the following reasons. First, PTA has been shown to have better predictive ability with clinical outcomes compared with GCS [15-18]. Many patients are oriented by the time they are first assessed, and therefore achieve the top score on the GCS scale, which leads to the common misperception that a perfect score means a normal examination [62]. Ponsford and colleagues conceptualised the Revised Westmead PTA Scale for screening of PTA in patients with mTBI, identifying it as more sensitive to the presence of PTA than GCS [63]. Shores and colleagues [17] studied the diagnostic accuracy of the Revised Westmead PTA scale and showed that at the time of the second neurological observation 60% of participants were not able to lay down new memory; yet 87% of them had been assigned a GCS of 15. Another recent study [15] showed that whereas patients with GCS scores of 13 or 14 did not differ from those with a score of 15 with respect to neuroimaging abnormalities, patients who had experienced PTA for more than 30 minutes were more likely to have intracranial abnormalities on imaging. So, by using a validated tool as part of the neuro-observations until patients receive an optimal score, one reduces the risk of failing to classify mTBI patients and prevents patients from being discharged from hospital while they are suffering from acute cognitive impairment [19,20].

Second, this outcome can be reliably measured (the score is an objective measure in the medical records). Finally, given the estimated low rate of appropriate PTA screening (see sample size section), there is considerable opportunity for improvement.

#### Secondary outcomes

##### Clinical practice outcomes

Two other measures for PTA were selected as secondary outcomes. ‘PTA screening-tool’ measures whether the administration of the validated tool was completed at least once. ‘Memory-clinical’ measures whether staff members have made an assessment of memory using questions in their clinical assessment. Other secondary measures assessing the effectiveness of the intervention in improving the ED management of mTBI are listed in Table 3. On the cohort of patients for whom risk criteria are recorded, ‘CT scan-clinical criteria’ (CT) measures whether a CT scan was provided in the presence of a risk factor that justifies the scan (see Additional file 1). This measure is therefore indicating whether a scan was appropriately received; however, we will not be able to measure whether a scan was ‘appropriately denied’ because of the inconsistency in recorded clinical criteria. ‘Provision of patient information’ (INFO) measures whether information was recorded as provided upon discharge home from the ED. Via the chart audit, we will not be able to measure what information was handed out (for example, the information used in our intervention [40] or other). However, this will be assessed in the patient follow-up interview in NET-Plus hospitals. Two measures of ‘safe discharge’ are included, the first being a composite score of PTA and INFO (for all patients), and the second provides a composite measure of whether the patient received appropriate care for all of the three clinical practices (PTA, CT, INFO; for the subgroup of people for whom risk criteria were recorded).

Clinical practice outcomes will be measured retrospectively through chart audit by an independent, trained chart auditor. Data will be collected over the 2 month period following the last intervention component.

##### Proxy measures of clinical practice

Secondary outcomes also include proxy measures of the clinical practices of interest. These proxy measures consist of self-report measures and clinical vignettes [64].

##### Predictors of clinical practices

Table 4 shows the factors we hypothesise to mediate the intervention effects, listed per clinical practice. Factors have been grouped into two categories: 1) collective level constructs – clinicians’ self-reported cognitions about their EDs team climate for innovation [65,66] in relation to the clinical management of mTBI in general; and 2) individual level constructs – clinicians’ self-reported cognitions in relation to each clinical practice (such as the extent to which the clinicians feel confident in screening for PTA using a validated tool (beliefs about capabilities), whether a clinician believes these clinical practices will lead to favourable outcomes (beliefs about consequences) [67], and whether a clinician intends to perform the practices) [68]. For our primary outcome, individual level predictors along the causal pathway will be assessed (see Table 4). We have decided to only include these for the primary outcome, and not other outcomes for two reasons: 1) because the primary outcome is the best measure of clinical practice from the medical records which can be measured for all patients; and 2) to reduce the responder burden for clinicians.

**Table 4:** Predictors of clinical practices

	**Data collection method**	**Outcome assessment period/timing**	**Data source**	**Level data collected**	**Analysis level**
**PTA, CT, INFO**					
**Cognitions in relation to mild traumatic brain injury related team climate**					
Team climate					
• Participative safety	Staff questionnaire (4-item)	Baseline/Endpoint	Doctors	Staff member	ED
			Nurses		
• Support for innovation	Staff questionnaire (3-item)	Baseline/Endpoint	Doctors	Staff member	ED
			Nurses		
• Vision	Staff questionnaire (4-item)	Baseline/Endpoint	Doctors	Staff member	ED
			Nurses		
• Task orientation	Staff questionnaire (3-item)	Baseline/Endpoint	Doctors	Staff member	ED
			Nurses		
**Behaviourally specific cognitions**					
Intention to adhere to recommended practice					
• PTA	Staff questionnaire (1-item)	Baseline/Endpoint	Doctors	Staff member	Staff member
			Nurses		
• CT	Staff questionnaire (1-item)	Baseline/Endpoint	Doctors	Staff member	Staff member
• INFO	Staff questionnaire (1-item)	Baseline/Endpoint	Doctors	Staff member	Staff member
			Nurses		
**PTA only**					
• Knowledge	Staff questionnaire (2-item)	Baseline/Endpoint	Doctors	Staff member	Staff member
			Nurses		
• Beliefs about capabilities	Staff questionnaire (3-item)	Baseline/Endpoint	Doctors	Staff member	Staff member
			Nurses		
• Beliefs about consequences	Staff questionnaire (3-item)	Baseline/Endpoint	Doctors	Staff member	Staff member
			Nurses		
• Social influences	Staff questionnaire (2-item)	Baseline/Endpoint	Doctors	Staff member	Staff member
			Nurses		
• Environmental context and resources	Staff questionnaire (3-item)	Baseline/Endpoint	Doctors	Staff member	Staff member
			Nurses		

CT, CT scan-clinical criteria; INFO, provision of patient information; PTA, appropriate PTA screening.

Proxy measures of clinical practice and predicting factors are measured using staff questionnaires. The NET team will prepare the questionnaire packs (including invitation letters, explanatory statements and baseline questionnaires) and send these to a local survey coordinator, who will be tasked with the distribution of the packs to their randomly selected staff members (for example, via mail or pigeon hole). Non-responders will receive reminders verbally or via email, and will be sent a reminder pack [69]. Questionnaires will be available upon request, and from the website (http://www.netprogram.org.au/).

**Figure 2:**
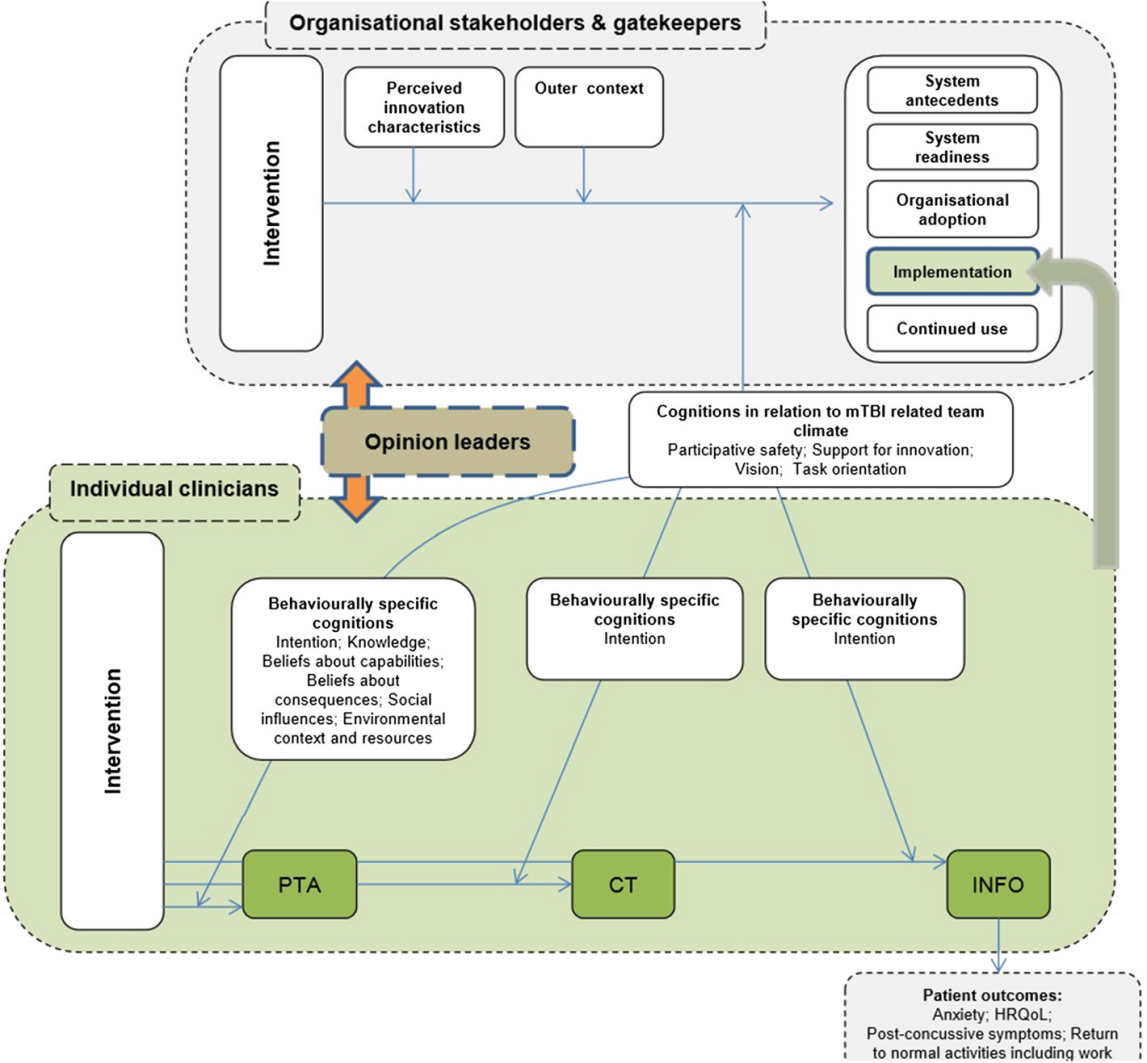
**Conceptual framework.** CT, CT scan-clinical criteria; HRQoL, health-related quality of life; INFO, provision of patient information; mTBI, mild traumatic brain injury; PTA, appropriate PTA screening.

Figure 2 depicts our conceptual framework, informed by Frambach and colleagues [70], Greenhalgh and colleagues [71] and Michie and colleagues [67]. It shows the specific factors our intervention will target, listed within categories (in bold).

##### Patient and cost outcomes

Table 5 outlines the patient and cost outcomes that will be included in the study. We hypothesise that provision of patient information containing advice, education, and reassurance upon discharge from the ED reduces anxiety and number of self-reported symptoms [40]. In addition, we will investigate whether this leads to improved post-accident functioning (return to normal activities including work and health-related quality of life (HRQoL)) and fewer adverse events (for example, re-presentations).

**Table 5:** Patient and cost outcomes

	**Data collection method**	**Outcome assessment period/timing**	**Data source**	**Level data collected**	**Analysis level**
**Clinical outcomes and quality of life**					
▪ Anxiety*	CATI questionnaire (7-item)	3-5 month post-discharge	Patient	Patient	Patient
▪ Post-concussive symptoms	CATI questionnaire (13-item)	3-5 month post-discharge	Patient	Patient	Patient
▪ Return to normal activities including work	CATI questionnaire (2-item)	3-5 month post-discharge	Patient	Patient	Patient
▪ Health-related quality of life	CATI questionnaire (12-item)	3-5 month post-discharge, data collected over last month	Patient	Patient	Patient
**Healthcare utilisation and costs**					
▪ Medical and surgical services received in ED/inpatient ward (including CT scan)	Chart audit	Retrospectively on a 2 month period post-intervention	Hospital record	Patient	Patient
▪ Re-presentation to ED	CATI questionnaire (1-item)	3-5 month post-discharge	Patient	Patient	Patient
	Chart audit	Retrospectively on a 2 month period post-intervention	Patient	Patient	Patient
▪ Healthcare visits in relation to mTBI (GP, brain clinical, other)	CATI questionnaire (3-item)	3-5 month post-discharge; data collected over last month	Patient	Patient	Patient
▪ mTBI-related medication use	CATI questionnaire (4-item)	3-5 month post-discharge; data collected over last month	Patient	Patient	Patient
▪ Direct costs delivering intervention (intervention group only)	Data abstraction surveys	On completion of delivery	Admin records	Intervention components	ED

*Primary outcome. CATI, computer-assisted telephone interview; CT, computed tomography; ED, emergency department; GP, general practitioner; mTBI, mild traumatic brain injury.

Patient outcomes in the NET-Plus hospitals will be collected via a computer-assisted telephone interview by a trained researcher, following consent received via a local ED staff member. Anxiety will be measured using the relevant questions in the Hospital Anxiety and Depression Scale [72,73]. Post-concussive symptoms will be measured using the 13-item Rivermead [74]. HRQoL will be measured using a 12-item short form health survey (SF-12) and SF-12-based SF6D [75,76]. The SF6D’s six domains - physical functioning, role participation, social functioning, bodily pain, mental health, and vitality - encompass variation in patient outcomes with respect to anxiety, interference with normal work and social function due to sleep problems, and physical functioning. Australian population norms for the SF-36-based SF6D are now available [77] and the SF6D has previously been used as a measure of HRQoL in TBI [78].

### Data quality assurance

Chart auditors will be trained to identify eligible patients in the medical record system, and to log data into the secure web-based system in participating hospitals. Auditors will assess the same pilot records, and training will continue until sufficient consistency is achieved. Auditors will receive a data collection manual with instructions (such as definitions and data dictionary). The web-based database will be designed to minimise errors (for example, inability to leave particular fields open, or warning when answer is outside expected range). Double data entry will be used for all paper-based questionnaires and returned questionnaires will be checked for consistent errors or missing data.

### Sample size

The primary outcome in the NET trial is appropriate PTA screening. In the calculation of sample size for this outcome, adjustment needs to be made for the clustered nature of the design. The variance inflation factor, or design effect, used to achieve this is a function of the average cluster size, variation in cluster size, and the intra-cluster correlation (ICC) [79].

The assumed ICC was calculated from two sources reporting ICCs from various datasets [80,81]. We included only ICCs for process measures in secondary care settings since empirical research has demonstrated that ICCs tend to be larger for 1) process measures compared with patient outcomes (median 0.063 versus 0.030, respectively), and 2) secondary care compared with primary care (median 0.061 versus 0.045) [63]. Thirty three ICC estimates for process measures in secondary care were available, and the median ICC was 0.18. The variation in cluster size was incorporated into the sample size calculation through an estimate of the coefficient of variation, defined as the ratio of the standard deviation of the cluster sizes to the mean cluster size [79]. The estimated coefficient of variation of 0.47 was calculated from annual attendance at EDs across 170 hospitals in Australia in 2009.

There were limited studies providing estimates of rates of compliance for our primary outcome [82], and no studies undertaken in adult populations. We therefore undertook a retrospective audit of two Australian metropolitan EDs. The estimated rates of appropriate PTA screening for patients presenting in these hospitals (with an initial GCS of 14 or 15) were 0% (95% CI 0% to 14%; n = 24) in one hospital, and 31% (95% CI 24% to 39%; n = 164) for a second (which had an mTBI protocol in place) (Bosch M, McKenzie J, unpublished observations). We expected that hospitals without a protocol for mTBI would be more interested in participating in the trial, and therefore calculated our sample size based on a control group rate of 10% (that is, closer to the estimated rate in the hospital with no protocol in place). We wish to detect an absolute increase in the rate of appropriate PTA screening of at least 20%. Our rationale for selecting a difference of 20% is based on considering the size of effect required to justify a resource intensive intervention such as NET, and estimates of the effects of local opinion leaders on professional practice observed across a range of studies [83].

To detect an absolute increase of 20% in the rate of appropriate PTA screening (equivalent to an odds ratio of 3.9, log odds 1.3) (assuming a control group rate of 10%, an ICC of 0.18, coefficient of variation = 0.47, an average of 30 patient participants per ED, and a 5% significance level) with 80% power, we will require 15 EDs per intervention group. A total of 30 EDs will provide 900 patient participants for whom ED staff management will be assessed. For 900 patient participants, the width of the 95% confidence interval for the observed difference in appropriate PTA screening rates between groups will be approximately ±14% (equivalent to a width of ±0.51 on the log odds scale). Allowing for 10% attrition in EDs, we plan to initially recruit 17 EDs per intervention group.

Power is dependent on the selected parameters, but in cluster trials is primarily determined by the chosen ICC and control group rate. Assuming all other parameters are as defined above, a lower control group rate (for example, 5%) will result in greater power (87%), while a higher control group rate (for example, 30%) would result in less power (60%). A smaller ICC (for example, 0.10) would result in greater power (94%), while a larger ICC (for example, 0.30) would result in less power (59%).

Sample size calculations were undertaken using the module *clustersampsi*[84] implemented in the statistical package Stata (StataCorp LP, USA) [85].

### Effectiveness analysis

The effectiveness of the intervention on management outcomes, predictors of management outcomes, and patient outcomes will be estimated with marginal modelling using generalised estimating equations. These models will appropriately account for correlation of responses of individuals within EDs. We plan to fit an exchangeable correlation structure where responses from the same ED are assumed to be equally correlated [86]. We will use robust variance estimation which yields valid standard errors even if the within-cluster correlation has been incorrectly specified [87,88]. For binary outcomes, a logit link will be used.

**Figure 3:**
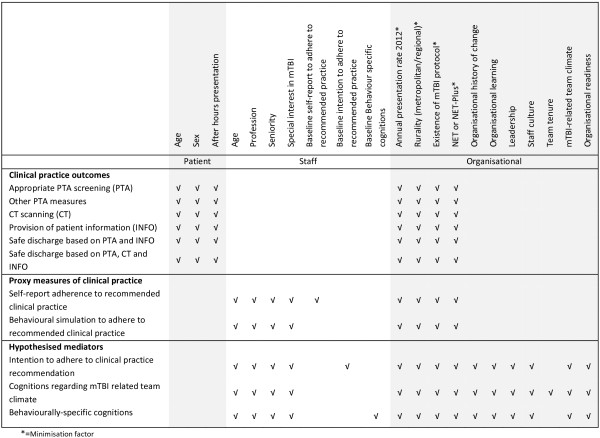
**Confounders.** mTBI, mild traumatic brain injury; NET, Neurotrauma Evidence Translation.

Models will include adjustment for minimisation factors (size (annual presentation rate in 2012), existence of an mTBI protocol, NET or NET-Plus, and rurality) and pre-specified potential confounding variables (Figure 3). The potential confounding variables have been selected through discussion amongst the investigators and from published research. All pre-specified confounders will be included in the models even when no baseline imbalance exists. In addition, for continuous outcomes which are collected at both baseline and follow-up (for example, clinicians’ self-reported cognitions, social and environmental influences in relation to the key behaviours, cognitions about their organisation and team climate) we will include the baseline measure of the outcome in the model. Adjustment for the baseline measure of a continuous outcome yields unbiased estimates of intervention effect in circumstances where there is baseline imbalance, and has the benefit of providing the most powerful analysis [89]. Our primary effectiveness analysis will be the model (as described above) that estimates the intervention effect on the primary outcome, appropriate PTA screening.

Estimates of intervention effect from these models with binary outcomes will yield odds ratios. To aid interpretability, we plan to also provide estimates of risk differences [90]. Estimates of risk differences will be computed from marginal probabilities estimated from the fitted logistic models [91].

Missing data for the clinical practice outcomes are likely to be minimal since eligible patients will be identified and data extracted through chart audit. Missing data could arise if EDs choose to withdraw post-randomisation but prior to data being extracted from clinical records, resulting in empty clusters. Accounting for empty clusters requires strong assumptions to be made about patient characteristics and outcomes based on ED staff or ED characteristics. We will attempt to examine the potential impact of empty clusters on the intervention effects for the clinical practice outcomes using weights to allow for patterns of ‘missingness’ [92]. For continuous outcomes, generalised estimating equations yield unbiased estimates of intervention effect when 1) data are missing completely at random, or 2) when the correlation structure is correctly specified and known covariates that are associated with the missing data mechanism are included in the model (missing at random) [93,94]. For the continuous proxy measures of clinical practice and hypothesised mediators, we plan to identify potential predictors of missing data through modelling (for example, [95]), and include these predictors in the primary analysis model. We will investigate methods to impute missing outcome data collected at baseline (for example, baseline behavioural constructs) [96].

All estimates of intervention effect will be reported with 95% confidence intervals. No adjustment will be made for multiple testing. All tests will be two-sided and carried out at the 5% level of significance. A full statistical analysis plan will be developed and written prior to undertaking any data analysis.

Potential confounders at patient level are collected via chart audit. Potential confounders at the level of staff members, as well as organisational history of change [97], organisational learning [98], leadership [99], staff culture [99] and team tenure [100] will be collected via self-report surveys. ED structural characteristics will be measured via a telephone checklist completed by the ED Director (or delegate).

### Process evaluation

Our theory-based evaluation of factors along the proposed causal pathway (see Table 4 and Figure 2) will be complemented by other components that form part of our process evaluation [101,102]; these are: 1) an assessment of intervention fidelity (whether the intervention was successfully and consistently delivered as planned (delivery), and whether it reached the target group (receipt)) [103,104]; 2) perceptions of success of delivery and receipt [105], and factors that contributed to this [103]; and 3) perceptions of acceptability and feasibility of the roll-out of the intervention [106].

Table 6 summarises the main elements included in the evaluation, methods planned, and indicative measures. A more detailed description of the fidelity assessment will be provided in a future publication.

**Table 6:** Overview of mixed-methods process evaluation

**Element**	**Method**	**Indicative measures/details**
Theory-based evaluation of factors along the proposed causal pathway	Quantitative	**Intervention and control group**: scales in endpoint surveys (see Table 4)
Delivery of intervention components	Mixed	**Intervention group**: detailed assessment of NET intervention delivery
		Local opinion leaders: researcher-assessed presence of both opinion leaders in each hospital for entire intervention delivery duration
		Stakeholder meeting: attendance of providers; delivery of messages and so forth (researcher assessed)
		Train-the-trainer: observer assessment of intervention components
		Local educational workshops: education sessions provided assessed via log-books completed by the clinical opinion leaders
		Materials and tools: availability assessed via self-report clinical opinion leaders and staff
		**Control group**: high-level assessment of potential delivery of (non-NET) intervention components assessed via a brief telephone interview with ED Director or delegate (for example, was someone in your ED championing improvements/providing education around the management of mTBI patients?)
Receipt and acceptability and feasibility of intervention elements	Mixed	**Intervention group**:
		Local opinion leaders: inclusion ‘local opinion leaders’ scale (ORCA) in staff endpoint surveys; staff perceptions of ‘availability’ and ‘credibility’ (in semi-structured interviews)
		Stakeholder meeting: attendance of key-stakeholders; acceptance of messages (both researcher assessed)
		Train-the-trainer: attendance of local opinion leaders assessed via attendance lists; participant assessment of acceptance of components (participant sheets)
		Local educational workshops: attendance of local staff assessed via attendance lists; participant acceptance of sessions provided, assessed via scales in endpoint surveys and semi-structured evaluation interviews
		Materials and tools: availability assessed via self-report of local opinion leaders and staff
Perceptions around successful implementation	Qualitative	**Intervention group**: Perceptions around the success of the implementation assessed in semi-structured interviews
Perceptions of factors influencing successful implementation	Mixed	**Intervention group**: Perceptions around factors influencing the implementation processes (for example, organisational readiness; perceived leadership support; perceptions around quality and clarity of evidence and recommendations and so forth, measured in semi-structured interviews)
		Inclusion of ‘leadership’ scale (ORCA) in staff endpoint surveys
Perceptions of acceptability and feasibility intervention as ‘package’	Mixed	**Intervention group**: Perceptions around usefulness and feasibility of roll-out measured in semi-structured interviews

ED, emergency department; mTBI, mild traumatic brain injury; NET, Neurotrauma Evidence Translation; ORCA, organisational readiness to change assessment.

We plan to conduct semi-structured interviews with ED Directors, local opinion leaders and staff members in eight intervention hospitals. We will sample for maximum variation (for example, size and location of EDs). The interview guide and analyses will be guided by a theory-based framework that was used in phase two of this project (the interview phase). Analyses and interviewing will be done concurrently to inform emerging themes. If data saturation has not been reached after conducting interviews in eight hospitals, we will add sites and continue sampling until data saturation [107].

In addition, a brief checklist-guided structured interview will be conducted by telephone with ED Directors or delegates to assess sustainability of the changes in intervention hospitals (for example, plans to integrate a validated tool into charts).

### Ethical review and informed consent

Pre-recruitment ethical approval of this trial protocol has been granted by Alfred Health Human Research Ethics Committee (approval Number 398/12). When participating hospitals have been identified, local ethics and research governance procedures will be completed. This will include a “letter of understanding”, which contains information around what the trial entails, what the expectations are from hospitals and researchers, and the process to authorise the independent chart auditor to undertake the data extraction from medical records. The document will be signed following recruitment, and prior to randomisation. It will still be possible for EDs to withdraw from the study at any time should they wish to do so.

Consent procedures will be in line with recent guidance regarding ethical issues in CRTs [54,108], and include the following.

#### For all hospitals

ED Directors will be asked to consent to: 1) ED trial randomisation (NET or NET-Plus); 2) delivery of the intervention to their ED; 3) permission to approach staff members for data collection purposes; 4) extraction of medical record information by the chart auditor for eligible patients over a 2 month period; and 5) permission to use this data for the current study and potentially future linked studies, with the understanding that all identifiable information will be removed, unless participants stated otherwise.

#### For NET-Plus hospitals

For the hospitals that opt to participate in the NET-Plus component, ED Directors are requested to, in addition to the points in the preceding paragraph, also consent to patients being contacted by telephone by an ED staff member post discharge to: 1) ask consent to participate in the telephone follow-up interview that will be performed by a trained researcher with experience in follow-up in this patient group; and 2) to allow the NET researchers to receive their contact details.

#### Staff members, intervention delivery

In cluster trials, it is impractical/impossible for researchers to obtain informed consent from every individual cluster member to receive the educational intervention before randomisation; therefore, we applied for a waiver for intervention delivery to staff members. To ensure the decision is in the cluster’s interest, ED directors will be asked to consult their staff members prior to their decision to participate in this study [54].

#### Staff members, data collection

For staff members selected to receive written questionnaires, we will use the implied consent principle (that is when we receive a completed questionnaire this will constitute informed consent). This will be clearly explained in the introduction to the questionnaires. Return of a blank questionnaire will be considered as refusal to participate. Staff members invited to the process evaluation interview over the telephone will be identified by the local opinion leaders, receive an invitation letter and opt in by returning a consent form to the researchers.

#### Patients

##### NET component

The NET component of the trial does not recruit patients. Data extraction from patient files will be performed by a trained chart auditor with clinical experience, who is familiar with several medical records systems, and who will sign a confidentiality agreement. This person will be authorised by each hospital to do the chart audit, and hence can be considered part of the circle of care at that hospital. We have sought a waiver for consenting patients to this part of the study for the following reasons. First, the chart auditor will identify the patients retrospectively. At this time they will confirm eligibility, and, for feasibilities reasons, also collect the outcome data. If consent from patients had been sought following confirmation of eligibility, this would have required substantial additional resources. In addition, this process may have introduced selection bias, whereby those patients choosing to participate may have differed from patients who declined. Furthermore, only routinely collected information is extracted, and the central research team will only have access to non-identifiable information, which maintains the privacy of the patients. Finally, the intervention is not being delivered at the level of the patient; patients are only affected indirectly by the study intervention and there is limited risk of the intervention affecting their interests adversely [54,109].

##### NET-Plus component

In NET-Plus hospitals, patients included in the chart audit who meet the initial inclusion criteria for telephone follow-up (see section: recruitment of patients for follow-up) are contacted by telephone by an ED staff member post discharge to: 1) ask consent to participate in the telephone follow-up interview that will be performed by a trained researcher with experience in follow-up in this patient group; and 2) to allow the NET researchers to receive their contact details. The NET team will then send explanatory letters to the patients who consented to the above, to provide them with the information regarding the follow-up interview. This letter explains participation in the interview is entirely voluntary. At the start of the actual interview, the patients will again be given the opportunity to opt out of the study, should they wish to do so.

### Confidentiality of data

Confidentiality of data will be ensured via the following processes. All information collected will be entered via a web-interface into a secure server database. Each user will have a unique username and will be assigned a role that will provide them with access to only the information they need to see. The database administrator will have access to the full data, and will sign a privacy statement acknowledging responsibilities and restrictions on handling identifiable information according to ethics guidelines.

#### Patient data, NET component

Independent chart auditors who have been authorised by the hospitals to collect data will be able to see and log identifiable information (for example, patient numbers and names) in the database. For data quality purposes, patient data need to be re-identifiable. However, this information will not be visible to study researchers who will only be able to see “study participant numbers” (a unique number automatically generated by our database system) and non-identifiable information.

#### Patient data, NET-Plus component

Only patients who provide full verbal consent to the ED staff member will be identifiable from information in the database by the study researchers. Identification of patients for NET-Plus is necessary for completion of the patient interview.

#### Staff data

Survey packs (including a participant identification number) will be prepared by NET staff, and will be distributed by a local ED staff member (survey coordinator) in each hospital. For follow-up purposes, data need to be re-identifiable. Therefore, the survey coordinator will have access to names as well as study participant numbers. Completed surveys will be returned directly to the researchers and the data will be entered into the database. Therefore, the survey coordinator will not have access to the data. Researchers will have access to the data and study participant numbers, but not identifiable information.

### Economic evaluation

A number of previous studies have estimated the costs and benefits associated with adherence to various diagnostic management strategies for mTBI [110-112]. Stein and colleagues [112] conducted a modelled cost-utility analysis comparing selective CT scanning based on the CCTHR, CT for all patients, skull radiography for all patients, prolonged ED observation, 24-hour hospital admission, and no treatment. Smits and colleagues [111] conducted a modelled cost-utility analysis based on data from the Computed Tomography in Patients with Minor Head Injury (CHIP) trial [26] (comparing selective CT as per the New Orleans Criteria, CCTHR, and CT in Head Injury Patients (CHIP) criteria) and CT for no patients. Holmes and colleagues [110] conducted a modelled cost-utility analysis comparing selective CT scanning (as per National Institute for Health and Care Excellence (NICE) guidelines, the CCTHR and various other guidelines), CT for all patients, and discharge for all patients without testing. Findings from each of these studies suggest that – at the mean – selective CT scanning is cost-effective in comparison to other strategies. However, in interpreting these findings, Holmes and colleagues [110] noted that “… the cost of CT scanning is very small compared to the estimated cost of caring for patients with brain injury worsened by delayed treatment” (page 1,423) and concluded that “… all hospitals receiving patients with minor head injury should have unrestricted access to CT scanning for use in conjunction with evidence based guidelines” (page 1,423). Comparisons between alternative criteria for selective CT suggested that they offer “broadly similar costs and quality-adjusted life years” [110] (page 1,428), though adherence to the medium risk CCTHR had the highest probability of being the most cost-effective strategy in the study conducted by Holmes and colleagues [110]. Results reported by Stein and colleagues [112] and Smits and colleagues [111] are broadly consistent with these findings.

The costs and benefits associated with adherence to various diagnostic management strategies are clearly relevant to the question: what is the cost-effectiveness of an implementation intervention to increase adherence to key recommendations regarding diagnostic management? However, addressing this question entails a number of complications that were not addressed in the studies by Stein and colleagues [112], Smits and colleagues [111] and Holmes and colleagues [110]. First, the potential for cost-effective implementation is predicated on the existence of an evidence-practice gap (see Table 1). Previously published studies by Stein and colleagues [112], Smits and colleagues [111] and Holmes and colleagues [110] set aside the difficulties associated with closing the gap between evidence-based recommendations and clinical practice. Statements of cost-effectiveness taken from these previous studies are therefore conditional upon the assumption of perfect adherence to each of the evaluated diagnostic management strategies [27]. Second, implementation typically entails an additional investment that may or may not be offset by any health gains and/or reductions in health service utilisation derived from increased adherence to evidence-based recommendations. The economic evaluation described here will be the first to consider the trade-off between the hypothesised increase in adherence due to implementation of key clinical EBCPG recommendations regarding the ED management of adult patients with mTBI, and the additional costs (savings) arising from the implementation intervention.

Specifically, economic evaluations alongside NET and NET-Plus will be conducted with the aim of quantifying additional costs (savings) and health gains arising from delivery of the NET implementation intervention in adult patients (18 years of age or older) with mTBI in Australia, compared with passive dissemination of the recommendations/EBCPG. Evaluation of costs and health gains arising from delivery of the intervention (*ex post* of development of the implementation intervention) will be informative to policy-makers and hospital administrators considering a wider roll-out of the NET implementation intervention. Secondary aims will be to determine whether the incremental treatment costs of the NET intervention are offset by reductions in health service expenditure (that is, whether implementation is cost-saving as compared with existing practice), and to determine whether the NET intervention dominates existing practice (that is, less costly but no less effective). The time horizons for inclusion of relevant costs and consequences for the trial-based evaluations described here coincide with the final scheduled follow-up of participants in NET (2 months post-intervention) and NET-Plus (3 to 5 months post-discharge for patients treated in the 2 months post-intervention). The economic evaluation alongside NET and NET-Plus will take a health sector perspective in identifying, measuring, and valuing costs and consequences within the time horizon for each component.

Additional methods for the economic evaluation including methods for the identification, measurement and valuation of outcomes and resource use are described in Additional file 2. Results from the economic evaluation alongside NET will be expressed as additional costs (savings) per patient appropriately screened for PTA, per patient who received patient information upon discharge home, and per patient safely discharged. Results from the economic evaluation alongside NET-Plus will be expressed as additional costs (savings) per point difference on anxiety questions of Hospital Anxiety and Depression Scale at 3 to 5 months post-discharge, additional costs (savings) per point difference on the Rivermead Post-concussive symptoms checklist, and additional costs (savings) per point difference in SF6D utility index scores.

## Discussion

The cluster trial described in this protocol aims to evaluate the implementation of a targeted theory- and evidence-informed intervention to improve key evidence-based recommended practices for the management of mTBI in Australian EDs. To our knowledge this is the first trial to evaluate this suite of key recommendations. It addresses calls to use and test theory-driven models of change from a range of scientific disciplines to enhance knowledge translation efforts in ED settings [113]. More broadly, we hope this protocol may assist those who are undertaking quality improvement studies in emergency care settings.

## Trial status

At the time of submission of this manuscript, recruitment of sites had been completed, and collection of baseline data had been started; however, data cleaning or analysis has not commenced. The trial was registered in the Australian New Zealand Clinical Trials Registry on 12 December 2012 (ACTRN12612001286831).

### Publication policy

The results from the trial will be published regardless of the outcome. Reporting of this trial will adhere to the relevant, and most up-to-date, CONSORT (Consolidated Standards of Reporting Trials) statement [90] and its relevant extensions [114-116].

## Abbreviations

CCTHR: Canadian Computed Tomography Head Rule; CRT: cluster randomised trial; CT: computed tomography; EBCPG: evidence-based clinical practice guideline; ED: emergency department; GCS: Glasgow Coma Scale; HRQoL: health-related quality of life; ICC: intra-cluster correlation; mTBI: mild traumatic brain injury; NET: Neurotrauma Evidence Translation; PTA: post-traumatic amnesia; TBI: traumatic brain injury.

## Competing interests

JMG is Editor-in-chief of *Trials*. Editorial decisions regarding publication of this manuscript were made independently by another editor. The remaining authors declare that they have no competing interests.

## Authors’ contributions

RLG and SEG were the lead investigators of the funding application and provided general oversight and input in the study design. MB co-led the design of the trial, designed the data collection instruments and contributed to the intervention, wrote the first draft of the manuscript and prepared the revised versions. JEM (project statistician) co-led the design of the trial and was responsible for the statistical aspects, and wrote the sections ‘randomisation and allocation concealment’, ‘sample size’ and ‘effectiveness analysis’. DM (project health economist) was responsible for the design of the economic evaluation, and wrote the ‘economic evaluation’ section. JJF, SEB, EJT and DAO contributed to intervention design and process evaluation. JLP contributed to the design of the intervention and the patient follow-up study. JVR contributed to the design of the patient follow-up study. JCK and AP contributed to intervention design and piloting of data collection tools. JMG provided critical review of drafts and contributed to revisions. All authors commented on a draft of the protocol and read and approved the final manuscript.

## Supplementary Material

Additional file 1 Overview of criteria for CT scanning in adults. Click here for file

Additional file 2 Additional methods for the economic evaluation. Click here for file
