# Supplementary material for: Implementing evidence-based recommended practices for the management of patients with mild traumatic brain injuries in Australian emergency care departments: study protocol for a cluster randomised controlled trial
Source: Trials. 2014 Jul 11;15:281. doi: 10.1186/1745-6215-15-281 (PMC4107995; doi:10.1186/1745-6215-15-281)
Supplement: Additional file 1 — Overview of criteria for CT scanning in adults. [file 1745-6215-15-281-S1.pdf]

NET-Trial additional File 1: overview of criteria for CT scanning in adults.

&	MAA NSW [1] and NSW ITIM [2]	SIGN [3]	NICE [4] and NZGG [5]	ACEP/CDC [6]	Canadian CT Head Rule (CCTHR) [7]	New Orleans Criteria (NOC) [8]	NEXUS-II criteria [9]	CT in Head Injury Patients (CHIP) criteria [10]
**Age**	√ (>65) #	√ (≥65) IF LOC/amnesia	√ (≥65) IF LOC/amnesia	√ (≥65)<br>**OR**<br>>60 IF LOC/amnesia	√ (≥65) IF LOC / amnesia / witnessed disorientation	√ (>60) IF LOC/amnesia	√ (≥65)	√ (≥60)<br>**OR**<br>*40-60 (minor^)*
**GCS score**	√ (<15 2 hrs post-injury)<br>**OR**<br>Deteriorating GCS<br>**OR**<br>Persistent abnormal alertness / behaviour / cognition	√ (<15 2 hrs post-injury **OR** after 1 hr of obs)<br>**OR**<br>Deteriorating level of consciousness	√ (<15 2 hrs post-injury)	√ (<15)	√ (<15 2 hrs post-injury) IF LOC / amnesia / witnessed disorientation	Their definition only included patients with GCS 15	Abnormal alertness / behaviour	√ (<15)<br>**OR**<br>Deteriorating GCS (≥2 points (1 hr after presentation)<br>**OR**<br>*Deteriorating GCS 1 point 1 hr after presentation (minor)*
**Amnesia**	Anterograde or retrograde >30 mins ([1] only)<br>**OR**<br>Persistent abnormal A-WPTAS score (<18 4 hrs post-injury)	√ (retrograde >30 mins)	√ (for events > 30 mins before impact)	√ (deficits in short term memory IF LOC/amnesia)	√ (before impact ≥ 30 mins) IF LOC / amnesia / witnessed disorientation	√ (deficits in short term memory - anterograde) IF LOC/amnesia		√ (PTA≥ 4 hrs)<br>**OR**<br>*persistent anterograde (minor)*<br>**OR**<br>*PTA of 2 to 4 hrs (minor)*
**Suspected skull fracture**	√ Clinical suspicion of skull fracture	√ (basal, depressed or penetrating)	√ (open or depressed **OR** signs of basal)	√ (physical signs of basilar)	√ (open or depressed **OR** any sign of basal) IF LOC / amnesia / witnessed disorientation		√	√ (Clinical signs)
**Vomiting**	√ (≥2) [1]<br>√ (especially if recurrent) [2]	2	√ (>1)	√	√ (≥2) IF LOC / amnesia / witnessed disorientation	√ IF LOC/amnesia	√ (persistent)	√
**Coagulopathy**	√ (e.g. Warfarin; alcoholic)	√ IF LOC/amnesia/ neurological feature	√ IF LOC/amnesia	√	Exclusion criterion for applicability rule	Not evaluated due to low numbers of patients with coagulopathy	√	√ (use of therapy)
**Post traumatic seizure**	√ (prolonged, focal or delayed)	√	√	√ IF LOC/amnesia		√ IF LOC/amnesia		√
**LOC**	√ (>5 mins)	√						*√ (minor)*
**Focal neurological deficit**	√	√	√	√			√	*√ (minor)*
**Headache**	√ (persistent, severe)	√ (severe and persistent)		√ IF LOC/amnesia<br>**OR**<br>severe		√ IF LOC/amnesia		
**Drug and alcohol intoxication**	√ (clinically obvious) [1] only			√ IF LOC/amnesia		√ IF LOC/amnesia		
**Trauma**	√ (multi-system) #<br>**OR**<br>Large scalp hematoma or laceration ([2] only) #			√ (visible, above clavicle IF LOC/amnesia)<br>(Multi-system trauma is exclusion criterion)		√ (visible, above clavicle) IF LOC/amnesia	√ (scalp hematoma)	
**Dangerous $ mechanism of injury**	√ #	√	√ IF LOC/amnesia	√	√ IF LOC / amnesia / witnessed disorientation			√<br>**OR**<br>*fall any elevation (minor)*
**Other**	√ (Known neurosurgery / neurological impairment) #<br>√ (Delayed presentation or re-presentation) #							*√ (contusion skull (minor)*

[1] notes that clinical judgement is required with these criteria; ^ [10] Minor = CT only indicated in the presence of at least 2 minor criteria

& Entry criteria / description of population

[1] Guideline relevant for adults who sustain a mechanical injury or blow to the head from external forces (excluding complicated closed head injury; moderate/severe TBI; acquired brain injury; cerebral haemorrhage; skull fracture). Criteria include: 1) one or more of the following: confusion or disorientation, LOC <30 mins, PTA <24 hrs, and/or transient neurological abnormalities e.g. focal signs, seizures, intracranial lesions not requiring surgery; 2) GCS 14 or 15 at 30 mins post-injury or later upon presentation for health care; 3) GCS 13 at 30 mins post-injury or later upon presentation for health care and a normal CT scan. These manifestations must not be due to alcohol, drugs, medications and caused by other injuries.

[2] Patients with initial GCS score of 14 or 15 on arrival at hospital following acute blunt head trauma (with or without a definite history of LOC or amnesia.

[3] Patients with a history of a blow to the head or the presence of a scalp wound or those with evidence of altered consciousness after a relevant injury. Mild defined as GCS 13 to 15.

[4] Any trauma to the head – other than superficial injuries. Mild defined as GCS 13 to 15.

[5] TBI defined as acute brain injury resulting from mechanical energy to the head from external physical forces. Operational criteria include: confusion or disorientation; LOC; PTA; other neurological abnormalities such as focal neurological signs, seizure and / or intracranial lesion. These manifestations must not be due to alcohol, drugs, medications and caused by other injuries. Mild defined as GCS 13 to 15.

[6] Inclusion criteria for relevance of guidance are: non-penetrating trauma to the head, presentation to the ED within 24 hrs of injury, GCS 14 or 15 on initial evaluation in ED, age 16 or greater. Exclusion criteria include: penetrating trauma; patients with multi-system trauma; GCS < 14 on initial evaluation in the ED, and age < 16 years.

[7] Minor head injury defined as witnessed LOC, definite amnesia, or witnessed disorientation in patient with GCS 13 to 15

[8] Minor head injury defined as LOC in patients with normal findings on brief neurological exam and GCS 15. LOC defined as witnessed or patient unable to remember the traumatic event.

[9] Blunt head trauma patients – sub-population 'minor': GCS 15

[10] Minor head injury patients defined as blunt injury to the head who have a normal or minimally altered level of consciousness on presentation (GCS 13 to 15 and max LOC 15 mins and PTA max 60 mins)

$ Definitions regarding dangerous mechanisms

[1, 2] Motor vehicle accident election / roll-over; pedestrians / cyclist hit by vehicle; falls > own height or five stairs; falls from horses / cycles etc.; focal blunt trauma, e.g. bat / ball / club.

[3] Pedestrian struck by motor vehicle, occupant ejected from vehicle, significant fall from height, or significant assault (e.g. blunt trauma with weapon)

[4, 5] Pedestrian struck by motor vehicle, occupant ejected from vehicle, or fall from height of greater than one metre or five stairs

[6] Ejection from motor vehicle, a pedestrian struck, fall from a height > 3 feet or 5 stairs

[7] Pedestrian struck by vehicle, occupant elected from motor vehicle, fall from elevation ≥ 3 feet or 5 stairs

[10] Pedestrian or cyclist versus vehicle OR ejected from vehicle

List of abbreviations

A-WPTAS, Abbreviated Westmead Post Traumatic Amnesia Scale; CT, Computed Tomography; ED, Emergency Department; GCS, Glasgow Coma Scale; hr(s), hour(s); LOC, Loss Of Consciousness; (m)TBI, (mild) Traumatic Brain Injury; mins, minutes; PTA, Post Traumatic Amnesia;

References

1. Motor Accident Authority New South Wales: **Guidelines for mild traumatic brain injury following closed head injury**. Sydney; 2008.
2. NSW Institute of Trauma and Injury Management: **Initial management of closed head injury in adults, second edition**: NSW Ministry of Health; 2011.
3. Scottish Intercollegiate Guidelines Network: **Early management of patients with a head injury. A national clinical guideline**: Edinburgh Scotland; 2009.
4. National Institute for Clinical Excellence: **Head injury. Triage, assessment, investigation and early management of head injury in infants, children and adults**. London, UK.
5. New Zealand Guidelines Group: **Traumatic Brain Injury: Diagnosis, Acute Management and Rehabilitation**. Wellington; 2006.
6. Jagoda AS, Bazarian JJ, Bruns JJ, Jr., Cantrill SV, Gean AD, Howard PK, Ghajar J, Riggio S, Wright DW, Wears RL *et al*: **Clinical policy: neuroimaging and decisionmaking in adult mild traumatic brain injury in the acute setting**. *Ann Emerg Med* 2008, **52**(6):714-748.
7. Stiell IG, Wells GA, Vandemheen K, Clement C, Lesiuk H, Laupacis A, McKnight RD, Verbeek R, Brison R, Cass D *et al*: **The Canadian CT Head Rule for patients with minor head injury**. *Lancet* 2001, **357**(9266):1391-1396.
8. Haydel MJ, Preston CA, Mills TJ, Luber S, Blaudeau E, DeBlieux PM: **Indications for computed tomography in patients with minor head injury**. *N Engl J Med* 2000, **343**(2):100-105.
9. Mower WR, Hoffman JR, Herbert M, Wolfson AB, Pollack CV, Jr., Zucker MI: **Developing a decision instrument to guide computed tomographic imaging of blunt head injury patients**. *J Trauma* 2005, **59**(4):954-959.
10. Smits M, Dippel DW, Steyerberg EW, de Haan GG, Dekker HM, Vos PE, Kool DR, Nederkoorn PJ, Hofman PA, Twijnstra A *et al*: **Predicting intracranial traumatic findings on computed tomography in patients with minor head injury: the CHIP prediction rule**. *Ann Intern Med* 2007, **146**(6):397-405.
