# Supplementary material for: Implementing evidence-based recommended practices for the management of patients with mild traumatic brain injuries in Australian emergency care departments: study protocol for a cluster randomised controlled trial
Source: Trials. 2014 Jul 11;15:281. doi: 10.1186/1745-6215-15-281 (PMC4107995; doi:10.1186/1745-6215-15-281)
Supplement: Additional file 2 — Additional methods for the economic evaluation. [file 1745-6215-15-281-S2.pdf]

NET-Trial additional file 2: additional methods for the economic evaluation

The intervention and comparator, perspective and time horizon for the economic evaluations alongside NET and NET-Plus are described in the main text. Additional methods for the economic evaluations including the identification, measurement and valuation of costs and consequences, adjustment for differential timing and methods for summarising decision uncertainty are described below.

Scope of the analysis

The shorter time-horizon for the NET component necessarily excludes costs and consequences beyond discharge except in-so-far as they are reflected in adherence to the key recommendations of the EBCPG. Likewise, costs and consequences occurring subsequent to the final follow-up for NET-Plus (three to five months post-discharge) will be excluded from the economic evaluation alongside NET-Plus. In addition, we exclude some dimensions of health and HRQoL not captured by the clinical practice outcomes (in the case of NET) or patient-level outcomes (in the case of NET-Plus) on the grounds that their inclusion would be unlikely to alter policy recommendations. Research and evaluation costs will be excluded except where they might plausibly contribute to a clinically significant treatment effect. Costs common and invariant to both intervention and control groups (*e.g.*, costs associated with development and standard dissemination of the guideline) will not be explicitly calculated for the incremental analysis described here. Finally, some cost categories unlikely to produce clinically and economically significant variation in incremental cost will be excluded (e.g. opportunity cost of patient time while attending treatment) to simplify our analysis [1].

Identification, measurement, and valuation of health outcomes

Pandor et al [2] identified a number of health benefits associated with adherence to evidence-based diagnostic management strategies in mTBI including reductions in mortality and morbidity from the timely identification and treatment of patients with neurosurgical lesions and patients with non-neurosurgical lesions whose condition subsequently deteriorates. Where the proportion of mTBI patients receiving CT scan under existing practice is lower than under the EBCPG (such that implementing the recommendations of the EBCPG would entail an increase in the proportion of patients receiving a CT scan), increased adherence will also entail the disbenefit of increased cancer risk due to increased exposure to ionising radiation [3]. The trial-based economic evaluation described here is not designed to quantify treatment effects and cost-effectiveness in terms of the final health benefits and disbenefits. For the economic evaluation alongside NET, we rely on evidence from previous studies (e.g. [4]) that the health benefits of adherence to selective CT scanning in accordance with various evidence-based guidelines outweigh the disbenefits of increased cancer risk (as compared to CT for all patients and discharge for all patients without testing) and treat adherence to the guideline as a surrogate for final health outcomes.

In line with the main analysis, the primary outcome for the economic evaluation alongside NET will be appropriate PTA screening of patients with mTBI (based on chart review). Secondary outcomes for the economic evaluation alongside NET will be 'INFO' and 'safe discharge' calculated per patient based on 'PTA', 'CT' and 'INFO'. For the economic analysis, intervention effects with respect to 'PTA', 'INFO', and 'safe discharge' will be estimated using methods specified for the main analysis.

For NET-Plus, economic evaluations will be conducted for measures of anxiety, post-concussive symptoms and HRQoL described in Table 5 of the main text; with intervention effects estimated using methods specified for the main analysis. Preference-based HRQoL weights from the SF12-based SF6D must be combined with a period of time to permit estimation of intervention effects in QALY terms (and incremental cost-effectiveness in cost per QALY terms). Completion of the SF12 is scheduled for a single time point at three to five months post-discharge. Where between-group differences exist regarding re-presentation to the ED and the timing of identification and management of patients with neurosurgical lesions and of patients with non-neurosurgical lesions whose condition subsequently deteriorates, any intervention effect with respect to HRQoL might also be expected to vary over time and, in particular, in the three to five months post-discharge. In the absence of repeated observation on HRQoL, we will treat measures of HRQoL as a summary measure of the effect of the intervention with respect to anxiety, sleep, functioning and adverse effects at a single point in time and we make no attempt to combine SF6D index scores with time for the purposes of calculating QALYs.

Identification, measurement, and valuation of resource use
Incremental costs will reflect resource use associated with delivery of the implementation intervention, and any changes in clinical practice and subsequent health effects that occur within the trial period. Cost items associated with delivery of the implementation intervention include administrative costs associated with coordinating the intervention activities described in Table 2 of the main text; labour/consumables required for delivery of local stakeholder meetings including attendance and preparation time for moderators, project officers and local stakeholders; labour and consumables required for train the trainer workshops; labour/consumables required for local ED training sessions; opportunity cost of venue for stakeholder meetings, train the trainer workshops, and local ED training sessions; production of local educational packs; and labour/consumables required for provision of tools and materials. Cost items associated with delivery of the control condition include administrative costs associated with coordinating the control activities described in Table 2; and labour/consumables required for distribution of the EBCPG to control EDs.

Costs arising from changes in clinical practice include the costs of clinical investigations including PTA screening and CT scanning. Previous modelled economic evaluations have also incorporated costs associated with increased hospital admission for observation on the basis that "…increased use of CT scanning for minor head injury (is) …associated with increased hospital admission" [4](p1424). Likewise, increased adherence to EBCPG may lead to increases in neurosurgical treatment and intensive care (i.e., more timely identification and management) as compared to existing practice [2].

Costs arising from changes in health status include the costs of primary care, over the counter and prescription pharmaceuticals, re-admission, and – in the case of temporary or permanent disability – the cost of rehabilitation and the use of paid/unpaid disability care. For the economic evaluation alongside NET, costs arising from changes in health status cannot be explicitly included in the cost analysis (as a consequence of the time horizon) but may be reflected in the 'surrogate outcome' of adherence to the key-recommendations of the EBCPG. In the case of permanent disability, the majority of costs associated with disability care will be excluded from the economic evaluation alongside NET-Plus (as a consequence of the time horizon). Other costs arising from changes in

health status will be captured in the economic evaluation alongside NET-Plus only if they occur within three to five months of discharge.

Resource use associated with the delivery of the implementation strategy will be estimated from administrative and financial records detailing costs associated with the production and distribution of materials; total person hours spent in organising and facilitating workshops and training sessions; duration of and attendance at workshops and training sessions; venue location and total hours venue use for workshops and training sessions. Time spent by local ED trainers and ED staff in local training sessions will be estimated based on self-report questionnaire as described in Table 5. Resource use associated with a change in clinical practice and subsequent health effects will be based on patient self-report in the three to five -month period post-discharge and based on chart audit for the initial presentation to the ED and any subsequent direct admission for hospital inpatient care.

Unit costs for health service resource use during inpatient care and post-discharge will be as per the Manual of Resource Items for use in submissions to the Commonwealth of Australia's Pharmaceutical Benefits Advisory Committee [5]. Unit costs for resource use (e.g. cost per CT scan, cost per PTA screening) within the ED will be based on Scheduled Fees for relevant MBS items and average hourly wage rates from relevant industry awards.

For the economic evaluation alongside NET, total cost per patient by ED will be obtained as the summation of delivery cost per patient by ED and the cost of medical and surgical services received in ED/inpatient ward by patient. For the economic evaluation alongside NET-Plus, total cost per patient by ED will be obtained as the summation of delivery cost per patient by ED, the cost of medical and surgical services received in ED/inpatient ward by patient, cost of health service use post-discharge by patient, and the cost of re-admissions by patient.

Given the characteristic distribution of health costs (truncated at zero and right skewed), the importance of obtaining readily interpretable marginal effects, and our interest in population-average effects, we will model intervention effects on total costs using one-part GEEs with a log link rather than transformed ordinary least squares or two-part models [6]. Specification of the log link for the GEE model permits natural interpretation of marginal effects on cost without retransformation [6]. Correlation structure, standard errors and controls for confounding variables will be as specified for the main analysis.

Uncertainty

Cost-effectiveness acceptability curves (CEACs) will be derived from the joint density of incremental costs (ΔC) and incremental effects (ΔE) for the intervention as compared to existing practice. The joint density will be obtained via non-parametric bootstrapping from the distribution of observed cost/effect pairs for patient participants. Separate CEACs will be derived for upper/lower bound estimates of a pre-specified set of uncertain parameters not estimated with sampling error: unit cost for ED staff time (AUD 0, average hourly wage rate) and the discount rate (3%, 7%).

References

1. Drummond M, Sculpher M, Torrance G, O'Brien B, Stoddart G: **Methods for the economic evaluation of health programmes**, 2nd edn: New York Oxford University Press; 1997.
2. Pandor A, Harnan S, Goodacre S, Pickering A, Fitzgerald P, Rees A: **Diagnostic accuracy of clinical characteristics for identifying CT abnormality following minor head injury: a systematic review and meta-analysis**. *J Neurotrauma* 2011.
3. Berrington de Gonzalez A, Mahesh M, Kim KP, Bhargavan M, Lewis R, Mettler F, Land C: **Projected cancer risks from computed tomographic scans performed in the United States in 2007**. *Arch Intern Med* 2009, **169**(22):2071-2077.
4. Holmes MW, Goodacre S, Stevenson MD, Pandor A, Pickering A: **The cost-effectiveness of diagnostic management strategies for adults with minor head injury**. *Injury* 2012, **43**(9):1423-1431.
5. Pharmaceutical Evaluation Section (PES): **Manual of resource items and their associated costs for use in major submissions to the Pharmaceutical Benefits Advisory Committee involving economic analyses (Version 4.0)**; December 2009.
6. Buntin MB, Zaslavsky AM: **Too much ado about two-part models and transformation? Comparing methods of modeling Medicare expenditures**. *J Health Econ* 2004, **23**(3):525-542.
